# A semi-supervised fracture-attention model for segmenting tubular objects with improved topological connectivity

**DOI:** 10.1093/bioinformatics/btaf013

**Published:** 2025-01-12

**Authors:** Yanfeng Zhou, Liqun Zhong, Zichen Wang, Yang Ge

**Affiliations:** School of Artificial Intelligence, University of Chinese Academy of Sciences, Beijing 100049, China; State Key Laboratory of Multimodal Artificial Intelligence Systems, Institute of Automation, Chinese Academy of Sciences, Beijing 100098, China; School of Artificial Intelligence, University of Chinese Academy of Sciences, Beijing 100049, China; State Key Laboratory of Multimodal Artificial Intelligence Systems, Institute of Automation, Chinese Academy of Sciences, Beijing 100098, China; School of Artificial Intelligence, University of Chinese Academy of Sciences, Beijing 100049, China; State Key Laboratory of Multimodal Artificial Intelligence Systems, Institute of Automation, Chinese Academy of Sciences, Beijing 100098, China; School of Artificial Intelligence, University of Chinese Academy of Sciences, Beijing 100049, China; State Key Laboratory of Multimodal Artificial Intelligence Systems, Institute of Automation, Chinese Academy of Sciences, Beijing 100098, China

## Abstract

**Motivation:**

Ensuring connectivity and preventing fractures in tubular object segmentation are critical for downstream analyses. Despite advancements in deep neural networks that have significantly improved tubular object segmentation, existing methods still face limitations. They often rely heavily on precise annotations, hindering their scalability to large-scale unlabeled image datasets. Additionally, current evaluation metrics are insufficient for effectively capturing segmentation fractures.

**Results:**

To address these challenges, we propose a semi-supervised fracture-attention model (SSFA) for tubular object segmentation. SSFA enhances connectivity, reduces fractures, and maintains volumetric accuracy. It outperforms state-of-the-art models in topological performance. Extensive experiments on four public datasets validate the effectiveness of SSFA. Furthermore, we introduce a novel evaluation metric, the fracture rate, which provides an intuitive and quantitative assessment of segmentation fractures.

**Availability and implementation:**

Our source code is available at http://github.com/Yanfeng-Zhou/SSFA.

## 1 Introduction

Ensuring connectivity and preventing fractures in tubular object segmentation is essential for various downstream analyses, including vascular hemodynamic analysis, endoplasmic reticulum reconstruction, road design and neural circuit reconstruction.

Tubular object segmentation has made significant progress with the development of deep neural networks (DNNs). Common approaches include introducing prior inputs or additional learning modules to capture structural information, such as DM++ (Banerjee *et al*. 2020) and DSCNet (Qi *et al*. 2023); utilizing topology-preserving loss functions to emphasize topological differences, such as clDice (Shit *et al*. 2021) and TopoLoss (Hu *et al*. 2019); and applying post-processing techniques to repair fractures, such as VNR (Dulau *et al*. 2023). DM++ (Banerjee *et al*. 2020) proposes a topological-priors-based dual-branch model for neuron segmentation that leverages topological priors generated using the discrete Morse algorithm, combining them with raw images as input. DSCNet (Qi *et al*. 2023) introduces dynamic snake convolution to effectively capture features of slender and tortuous local structures. clDice (Shit *et al*. 2021) proposes a skeleton similarity measure that enhances the accuracy of skeleton pixel predictions. TopoLoss (Hu *et al*. 2019) uses persistent homology to identify critical topological points in segmentation results, guiding the model to focus on these points. Boundary-Aware Loss (Ngoc *et al*. 2021) employs the Minimum Barrier Distance cut algorithm to detect segmentation boundaries and incorporate boundary information into DNNs. VNR (Dulau *et al*. 2023) proposes a post-processing pipeline for retinal vessels that reconnects eligible branches. (Mosinska *et al*. 2018) introduces an additional VGG (Simonyan and Zisserman, 2014) network to repair fractures in segmentation results.

These strategies have demonstrated promising results in reducing fractures in tubular object segmentation. However, they heavily rely on accurate manual annotations, which are laborious and time-consuming to produce. Consequently, these methods are often restricted to labeled images for fully supervised training.

In tubular object segmentation, connectivity is more critical than volumetric accuracy. Several topological evaluation metrics have been proposed, including β error (Hu *et al*. 2019), Euler characteristic (Shit *et al*. 2021), StreetMover Distance (Belli and Kipf, 2019), and APLS (Van Etten *et al*. 2018). While these metrics provide valuable insights, they fail to intuitively and effectively capture the connectivity and fractures in segmentation results.

In this study, we propose a semi-supervised fracture-attention model (SSFA) for segmenting slender tubular objects, leveraging a small number of labeled images alongside additional unlabeled images for consistency training. SSFA consists of two segmentation networks, each initialized with different parameters. We introduce the Fracture-Attention Map (FA Map) as an additional input to each network and use segmentation predictions to iteratively update the FA Map during training. This enables the model to focus on fracture-prone structures in tubular objects. Our model achieves state-of-the-art topological performance while preserving volumetric accuracy. Extensive benchmarking on four datasets demonstrates the effectiveness of our model. Additionally, we propose a novel evaluation metri, fracture rate (FR), which provides an intuitive and quantitative measure of fractures in tubular object segmentation results.

##  

**Figure 1.**
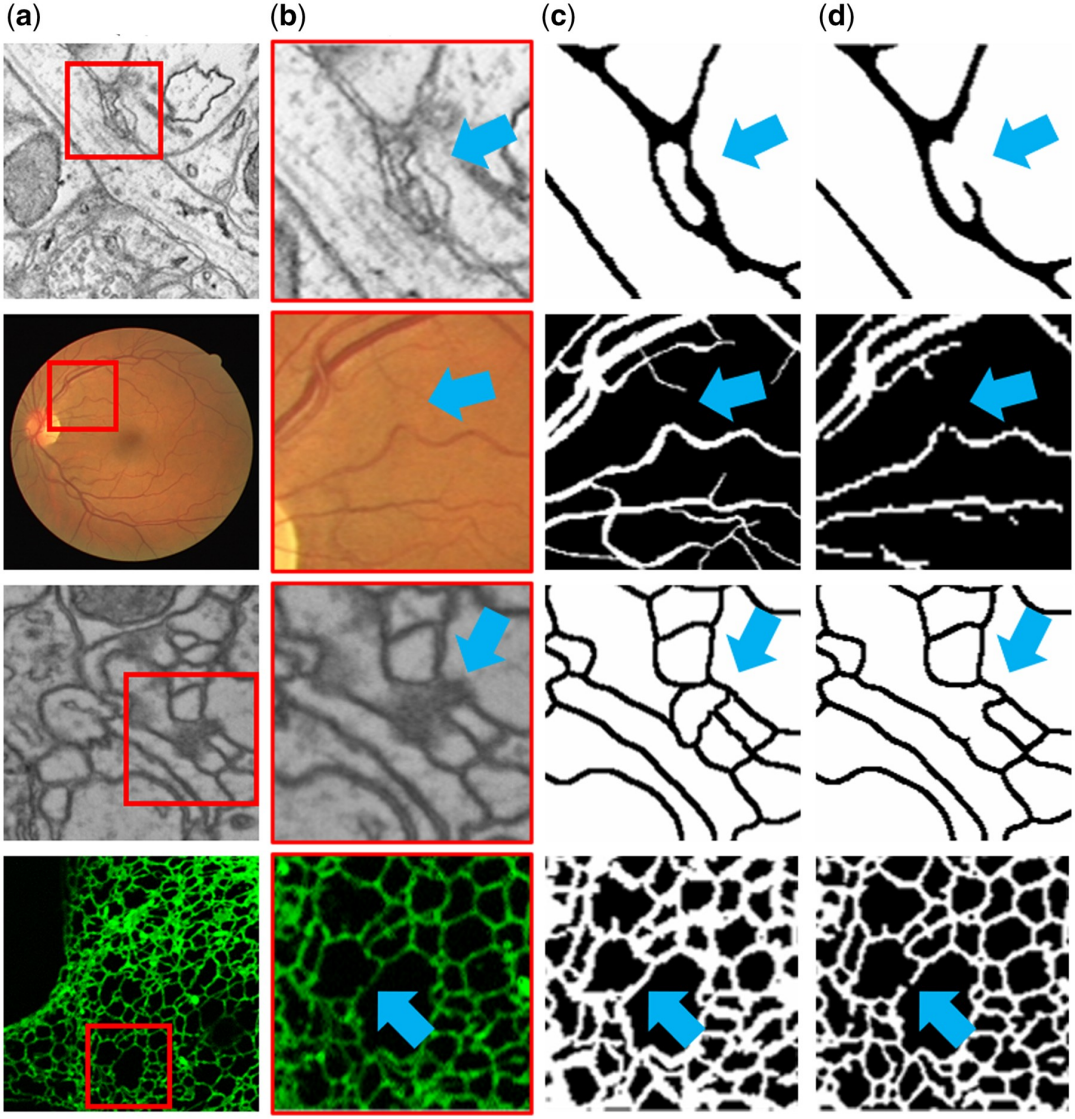
Using the SNEMI3D (SNEMI3D, Challenge 2013), DRIVE (Staal et al. 2004), CREMI (Funke et al. 2016), and ER (Luo et al. 2020) dataset, we visualize fractures in stenotic structures and semantically ambiguous regions. (a) Raw Images. (b) Stenotic structures and semantically ambiguous regions. (c) Ground truth. (d) Segmentation results of UNet (Ronneberger et al. 2015). Blue arrows highlight the fractures.

**Motivation**. Based on observations of the segmentation results of tubular objects, we find that fractures often occur in stenotic structures and semantically ambiguous regions (as shown in Fig. 1). To address this, we construct a Thickness-Sensitive Map (TS Map) and a Difference-Sensitive Map (DS Map) to guide the model’s focus on them. The TS Map emphasizes variations in the thickness of tubular structures, while the DS Map captures differences between segmentation predictions, which frequently correspond to areas with semantic ambiguity. By combining the TS Map and DS Map, we generate the FA Map. It serves as a prior input, guiding the model to concentrate on these fracture-prone regions and improving segmentation continuity.

## 3 Method

We provide an overview of the proposed model, SSFA, in Section 3.1. Then we introduce the generation and update of the FA Map in Section 3.2. We further introduce network architecture in Section 3.3. Finally, we introduce a novel metric for evaluating the FR in segmentation results in Section 3.4.

### 3.1 Overview

**Figure 2.**
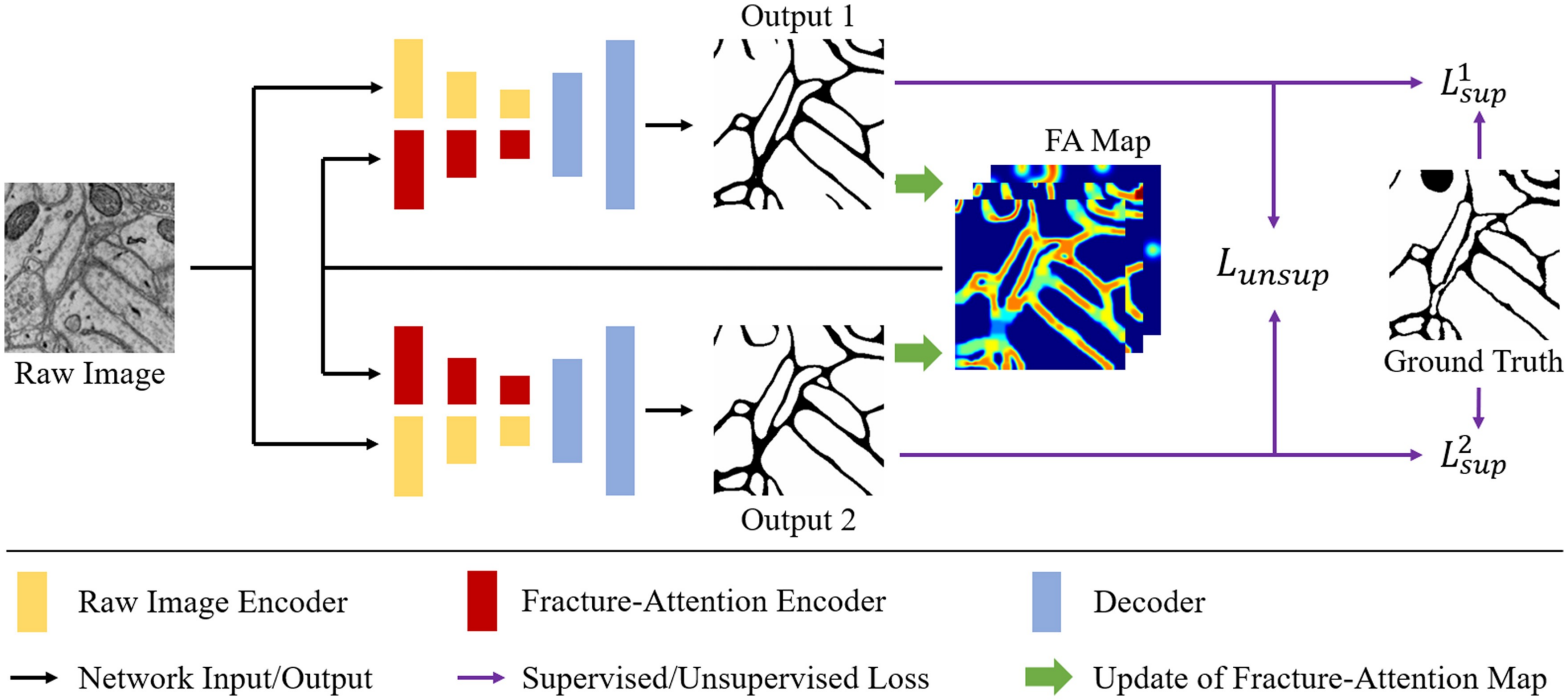
Overview of SSFA. It consists of two networks with the same architecture but different initialization parameters. Both networks take raw images and the FA Map as inputs. During training, the FA Map is iteratively updated based on the dual segmentation predictions, enabling the model to dynamically refine its focus on fracture-prone regions.

Figure 2 provides an overview of the SSFA model. It consists of two segmentation networks initialized with different parameters, both based on an encoder–decoder architecture. The encoder takes raw images and the FA Map as inputs to extract semantic information and identify fracture-prone structures. These features are then fused and passed to the decoder, which generates the segmentation predictions. We further elaborate on this encoder–decoder architecture in.

The differing parameters of the two networks result in consistent output variations, which are leveraged for semi-supervised training. During each training iteration, the dual outputs and their differences are used to iteratively update the FA Map. This mechanism enables the model to focus more effectively on structural changes and differences within tubular objects. The initialization and updating process of the FA Map is discussed in detail in Section 3.2.

Our model is optimized by minimizing supervised loss on labeled images and dual-output consistency loss on unlabeled images. The total loss $L_{\text{total}}$ is defined as follows:

(1) $$L_{\text{total}}=L_{\text{sup}}+\lambda L_{\text{unsup}},$$

where $L_{\text{sup}}$ is supervised loss, $L_{\text{unsup}}$ is unsupervised loss, i.e. the dual-output consistency loss, and λ is a weight that controls the balance between $L_{\text{sup}}$ and $L_{\text{unsup}}$. The weight λ increases linearly with training epochs:

(2) $$\lambda =\lambda_{\text{max}}*\frac{\text{epoch}}{\text{max}\_\text{epoch}},$$

where $\lambda_{\text{max}}$ is the maximum weight used to control the balance between $L_{\text{sup}}$ and $L_{\text{unsup}}$. We compare the performance of different $\lambda_{\text{max}}$ across various datasets in the ablation studies in Section 4.5. $L_{\text{sup}}$ is defined as:

(3) $$L_{\text{sup}}=L_{\text{sup}}^{1}(p_{i}^{1},y_{i})+L_{\text{sup}}^{2}(p_{i}^{2},y_{i}),$$

where $p_{i}^{1}$ and $p_{i}^{2}$ represent dual segmentation predictions for the ith labeled image, $y_{i}$ represents the ground truth of the ith image. The unsupervised loss $L_{\text{unsup}}$ is generated by CPS Chen *et al*. (2021) strategy: one prediction is used as a pseudo-label to supervise the other, and vice versa. $L_{\text{unsup}}$ is defined as follows:

(4) $$L_{\text{unsup}}=L_{\text{unsup}}^{1}(p_{j}^{1},{\hat{y}}_{j}^{2})+L_{\text{unsup}}^{2}(p_{j}^{2},{\hat{y}}_{j}^{1}),$$

where $p_{j}^{1}$ and $p_{j}^{2}$ represent the dual segmentation predictions for the jth unlabeled image, and ${\hat{y}}_{j}^{1}$ and ${\hat{y}}_{j}^{2}$ represent the pseudo-labels generated by $p_{j}^{1}$ and $p_{j}^{2}$, respectively.

In this study, $L_{\text{sup}}^{1}(\cdot )$, $L_{\text{sup}}^{2}(\cdot )$, $L_{\text{unsup}}^{1}(\cdot )$ and $L_{\text{unsup}}^{2}(\cdot )$ all use the Dice loss Milletari *et al*. (2016). The complete training process and update iteration for the FA Map are outlined in Algorithm 1.

For the inference process, our model employs a two-stage inference. Specifically, we first initialize the FA Map corresponding to the input image. We use the initial FA Map and the image as input to generate the initial segmentation prediction. Based on this initial prediction, we update the FA Map. We use the updated FA Map, along with the input image, to generate the second-stage prediction. We select the second-stage prediction of the branch that performs better during training as the final segmentation result.

Algorithm 1. Training process

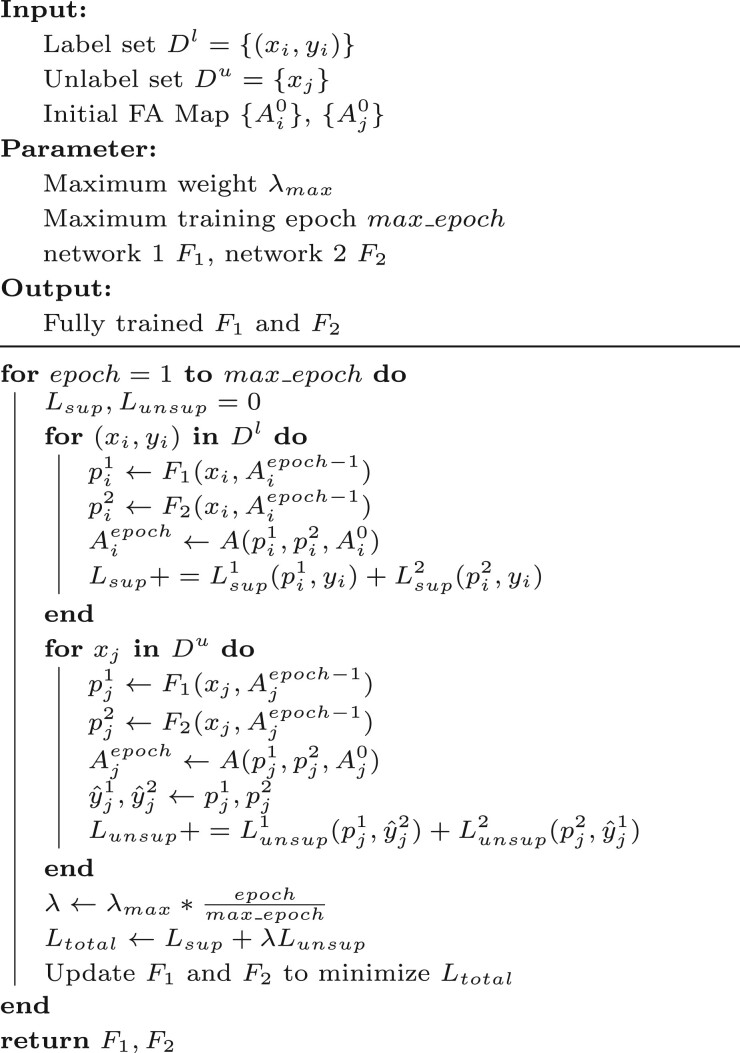

### 3.2 Fracture-Attention Map

**Figure 3:**
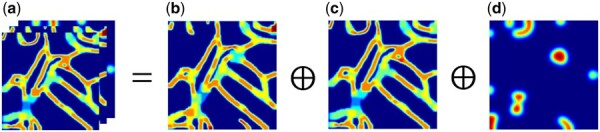
Composition of the FA Map. (a) FA Map. (b) TS Map 1 (generated by output 1). (c) TS Map 2 (generated by output 2). (d) DS Map. ⊕ represents channel concatenation.

We use the FA Map as an additional input to emphasize fracture-prone structures. Based on prior experience and observations, fractures often occur in slender and narrow branches or in semantically ambiguous regions. For slender and narrow branches, we construct TS Map 1 and TS Map 2 to emphasize variations in the thickness of tubular structures in segmentation predictions; For semantically ambiguous regions, where significant inconsistencies between segmentation predictions are observed, we construct the Difference-Sensitive Map (DS Map) to capture these differences. Thus, as illustrated in Fig. 3, the FA Map is composed of TS Map 1, TS Map 2, and DS Map. Their generation, update and initialization are as follows:

**Generation.** The generation of TS Map is shown in Algorithm 2:

1. Use the segmentation prediction P to generate the corresponding skeleton image S and distance mapping D.
2. Identify the maximum distance $d_{\text{max}}$ and minimum distance $d_{\text{min}}$ in D.
3. Using $d_{\text{max}}$, $d_{\text{min}}$ and the given attention level L, calculate the distance threshold $t_{l}$ for the l-th level of the TS Map.
4. Identify pixels in D with values smaller than $t_{l}$ and set the corresponding pixels in P to 0, resulting in $P_{l}$.
5. Use $P_{l}$ to generate the corresponding skeleton image $S_{l}$.
6. Perform difference matching using P, $P_{l}$, $S_{l}$, $S_{l-1}$ to generate the TS Map for the lth level.
7. Combine the TS Map from each level using pixel-wise addition to produce the final TS Map.

where L represents the total number of attention levels. A larger L provides finer division of attention levels. In this study, we set L=10 for all experiments.

Algorithm 2. Generation of TS Map

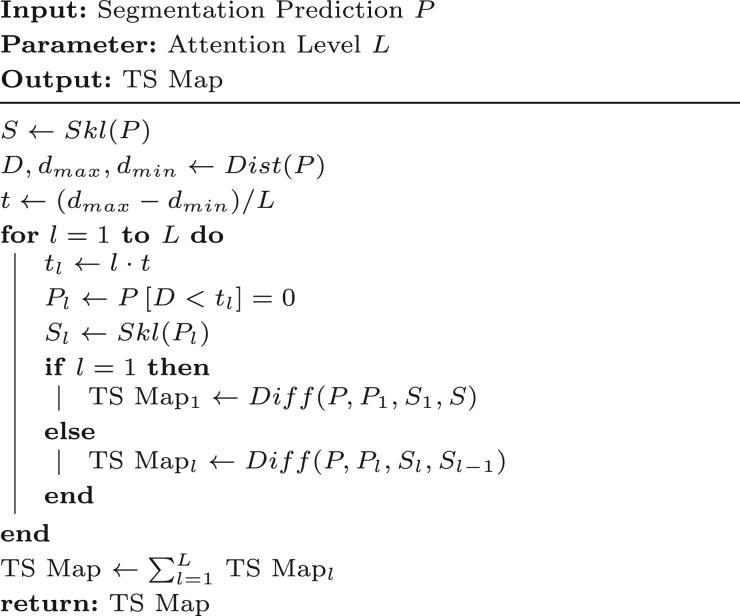

Algorithm 3. Generation of DS Map

 **Input:** Segmentation Prediction $P^{1}$, $P^{2}$

 **Output:** DS Map

 $S^{1}\leftarrow \mathrm{Skl}(P^{1})$

 $S^{2}\leftarrow \mathrm{Skl}(P^{2})$

 DS Map $\leftarrow \mathrm{Diff}(P^{1},P^{2},S^{1},S^{2})$

 **return:** DS Map

Algorithm 3 illustrates the generation of DS Map:

1. Use the segmentation predictions $P^{1}$ and $P^{2}$ to generate the corresponding skeleton image $S^{1}$ and $S^{2}$.
2. Use $P^{1}$, $P^{2}$, $S^{1}$ and $S^{2}$ for difference matching to generate the DS Map.

**Update.** To better capture structural changes and fracture-prone branches, we iteratively update the FA Map during the training stage. The FA Map at the rth epoch, denoted $A^{r}$, is defined as follows:

(5) $$A_{i|j}^{r}=(1-k)\cdot A_{i|j}^{0}+k\cdot {\hat{A}}_{i|j}^{r},$$

where $A_{i|j}^{0}$ represents the initial FA Map, ${\hat{A}}_{i|j}^{r}$ represents FA Map generated by the segmentation prediction at the rth epoch. k represents the update weight of $A_{i|j}^{r}$. Specifically, k=0 means the FA Map is not updated, while k=1 means ${\hat{A}}_{i|j}^{r}$ is directly used as $A_{i|j}^{r}$. We compare the performance of different values of k in the ablation studies in Section 4.5.

**Initialization.** For semi-supervised segmentation, the training dataset consists of both labeled and unlabeled images. We use the labeled images to train a fully supervised UNet (Ronneberger *et al*. 2015) to generate $A_{i|j}^{0}$.

### 3.3 Encoder–decoder architecture

**Figure 4.**
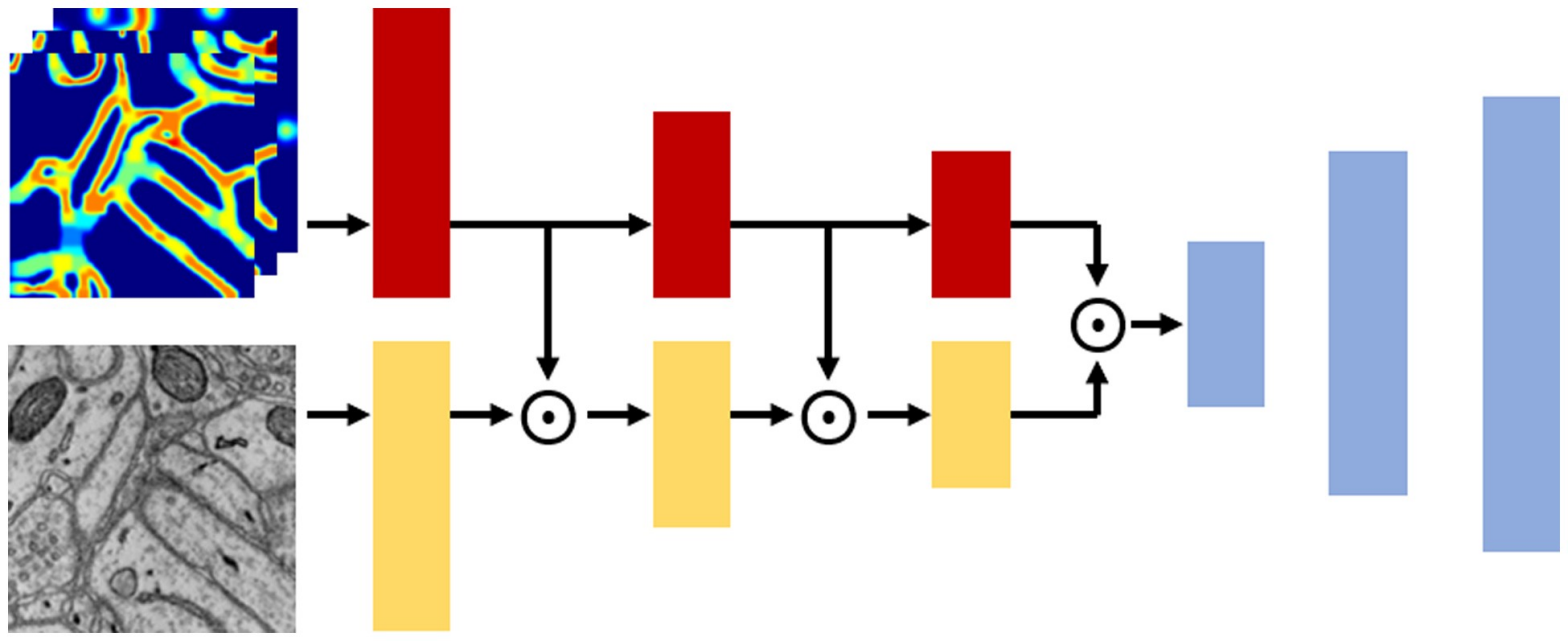
The encoder–decoder architecture of our model. Red and orange represent the FA Encoder and Raw Image Encoder, respectively. Blue represents the Decoder. ⊙ represents element-wise multiplication.

Figure 4 illustrates the encoder–decoder architecture of our model, which is based on UNet (Ronneberger *et al*. 2015). Different from UNet, our architecture introduces an additional encoder branch. Specifically, the encoder comprises two branches: the Raw Image Encoder and the FA Encoder. The Raw Image Encoder extracts semantic features from raw images, while the FA Encoder leverages the FA Map to help the model focus on stenotic structures and semantically ambiguous regions. To enhance feature fusion, we incorporate an attention mechanism Hu *et al*. (2018): before each downsampling step, we perform element-wise multiplication between the features from the two encoders. We demonstrate the effectiveness of this design in ablation studies in.

### 3.4 Fracture rate

**Figure 5.**
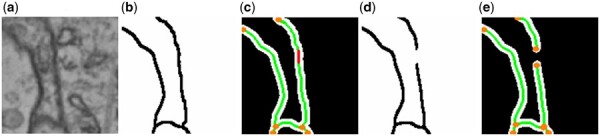
Visualization of fractures in segmentation results. (a) Raw image. (b) Ground truth. (c) Topological graph of the ground truth. (d) Segmentation result. (e) Topological graph of the segmentation result. Green lines represent edges, yellow dots represent nodes, red lines represent fractures.

Figure 5 illustrates the fractures in the segmentation results. To intuitively reflect these fractures, we propose a novel evaluation metric, FR, defined as follows:

(6) $$\text{FR}=\frac{N_{F}}{N_{Y}}\times 100\%,$$

where $N_{Y}$ represents the number of edges in the topological graph of the ground truth, and $N_{F}$ represents the number of fractured edges. The FR metric quantitatively evaluates the proportion of fractured edges relative to the total number of edges.

Algorithm 4. Calculation of FR

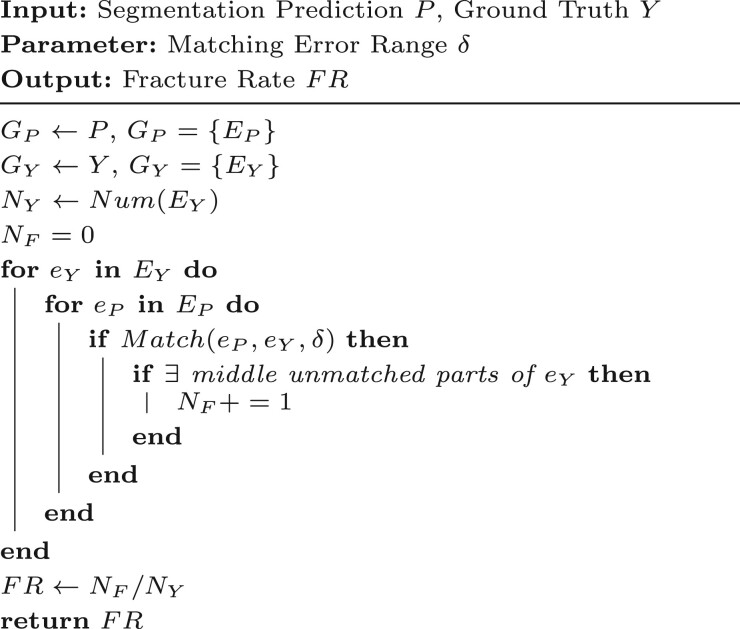

The calculation process for FR is detailed in Algorithm 4:

1. Use the segmentation prediction P and ground truth Y to generate the corresponding topological graphs $G_{P}$ and $G_{Y}$ (https://github.com/Image-Py/sknw).
2. Match the edges in $G_{Y}$ with those in $G_{P}$.
3. If after matching, there are still unmatched parts of the middle segment of edges in $G_{Y}$ within the error range δ, mark these edges in $G_{Y}$ as fractured.
4. Count the number of fractured edges $N_{F}$ and the total number of edges $N_{Y}$ in $G_{Y}$.
5. Calculate $FR=N_{F}/N_{Y}$.

where δ represents the matching error range. Since the skeletons of segmentation predictions and ground truth are not always identical, some differences between them are expected. Therefore, δ is used as the pixel overlap threshold for matching the topology between the segmentation predictions and the ground truth. In this study, we set δ=5 pixels for all experiments.

## 4 Experiments

### 4.1 Datasets

We evaluate our model on four public datasets with slender tubular objects: ER (Luo *et al*. 2020), SNEMI3D (SNEMI3D, Challenge 2013), CREMI (Funke *et al*. 2016), and STARE-DRIVE (Hoover *et al*. 2000, Staal *et al*. 2004).

**ER.** This dataset consists of fluorescence images for endoplasmic reticulum segmentation, including 223 images of size 256×256. We use 185 images for training and 38 images for testing.

**SNEMI3D.** This is a neuronal membrane segmentation dataset for electron microscopy images. It includes two image stacks. Each stack consists of 100 slices of size 1024×1024. We use the first stack for training and the second stack for testing.

**CREMI.** This is a neuronal membrane segmentation dataset for electron microscopy images. It includes three images stacks for three different types of neurons, with each stack comprising 125 slices of size 1250×1250. We use the first two stacks (250 slices) for training and the third stack (125 slices) for testing.

**STARE-DRIVE.** This dataset is used for retinal vessel segmentation and combines images from STARE (Hoover *et al*. 2000) and DRIVE (Staal *et al*. 2004). It includes 60 labeled images (20 from STARE, 40 from DRIVE) and 377 unlabeled images (from STARE). We use 30 labeled images and all unlabeled images for training, and the remaining labeled images for testing.

For CREMI and SNEMI3D, because the large image sizes are not convenient for training, we use a sliding window to crop the raw images to 256×256 to form a new training and test sets. Additional details on the experimental setup for these datasets are presented in Table 1.

**Table 1.** Experimental setup of four datasets.

Dataset	Modality	Annotation	# Train (# Labeled + # Unlabeled)	# Test
ER	Fluorescence microscopy	Endoplasmic reticulum	185 (37 + 148)	38
SNEMI3D	Electron microscope	Neuronal membrane	839 (191 + 648)	1600
CREMI	Electron microscope	Neuronal membrane	3575 (714 + 2861)	3075
STARE-DRIVE	Fundus retina	Retinal vessel	407 (30 + 377)	30

**Why choose them?** These datasets represent a range of scales, from hundreds to thousands, and cover diverse segmentation objects, including endoplasmic reticulum, neuronal membranes, and blood vessels. They are also widely used to evaluate segmentation performance on tubular objects (Hu *et al*. 2019, Shit *et al*. 2021, Yang *et al*. 2021), making the experimental results based on them both representative and convincing.

### 4.2 Evaluation metrics

For evaluating topological metrics, following Shit *et al*. (2021), Hu *et al*. (2019), Yang *et al*. (2021), we use β Error, $\beta_{0}$ Error, $\beta_{1}$ Error and clDice to evaluate performance. Additionally, we introduce our proposed metric, FR. To provide a comprehensive evaluation, we also report volumetric metrics, including pixel-wise accuracy (Acc), Dice and Jaccard.

### 4.3 Implementation details

We implement our model via PyTorch. Training and inference of all the models are performed on four NVIDIA GeForce RTX3090 GPUs. We use SGD with momentum to train models, the momentum is set to 0.9, and the weight decay is set to 0.00005. The batch size is set to 16. The number of epochs is set to 100. The initial learning rate is set to 0.1, and the learning rate decays by 0.5 every 25 epochs. Flip, rotation, transposition are used as data augmentation. During training and inference, input images are resized to 256×256.

### 4.4 Comparison with state-of-the-art models

We compare our model with previous state-of-the-art semi-supervised models, including MT (Tarvainen and Valpola 2017), CPS (Chen *et al*. 2021), XNet (Zhou *et al*. 2023), DCNet (Chen *et al*. 2023). Additionally, we evaluate fully supervised models, including UNet (Ronneberger *et al*. 2015), TopoLoss (Hu *et al*. 2019), clDice (Shit *et al*. 2021), and MRE (Huang *et al*. 2024).

As shown in Table 2, skeleton- or topology-based losses can enhance the performance of UNet, but they heavily depend on accurate annotations. Semi-supervised training with additional unlabeled images also has positive effects. In contrast, our model is specifically designed to address the thickness of tubular objects and highlight semantically ambiguous regions during semi-supervised training. This approach significantly reduces the occurrence of fractures and improves topological accuracy, while still maintaining volumetric accuracy.

**Table 2.** Comparison with state-of-the-art models on the ER, SNEMI3D, CREMI, and STARE-DRIVE test sets.^a^

Dataset	Method	Model	Topological					Volumetric		
			β↓	$\beta_{0}↓$	$\beta_{1}↓$	FR ↓	clDice ↑	Acc ↑	Dice ↑	Jaccard ↑
ER	Fully	UNet	39.35	23.95	15.40	10.67	93.39	90.89	83.14	71.14
		TopoLoss	22.80	17.15	5.65	9.34	94.39	91.45	84.74	73.51
		clDice	23.78	17.10	6.68	9.95	**94.74**	**91.66**	**85.19**	**74.21**
		MRE	**21.58**	**16.31**	**5.27**	**9.27**	93.78	91.24	84.61	73.32
	Semi	MT	37.00	22.30	14.70	11.37	93.81	91.07	83.66	71.91
		CPS	33.56	18.08	15.48	10.37	93.84	91.10	83.72	71.99
		XNet	35.00	22.45	12.55	10.41	93.75	90.85	83.44	71.59
		DCNet	47.06	35.08	11.98	11.78	92.02	90.28	81.97	69.45
		SSFA (Ours)	**19.23**	**14.65**	**4.58**	**9.21**	**94.71**	**91.56**	**85.33**	**74.42**
SNEMI3D	Fully	UNet	6.35	2.63	3.72	11.53	80.09	91.27	72.75	57.17
		TopoLoss	**2.14**	**1.73**	**0.41**	9.92	80.36	91.27	73.20	57.73
		clDice	2.66	2.10	0.57	11.44	**85.37**	90.38	73.67	58.31
		MRE	2.68	1.83	0.85	9.04	81.88	92.26	75.99	61.27
	Semi	MT	2.87	1.91	0.96	9.16	**84.69**	**92.50**	**76.20**	**61.56**
		CPS	2.71	1.90	0.81	**8.71**	84.57	**92.47**	**76.13**	**61.45**
		XNet	2.73	1.86	0.87	8.91	82.67	92.38	75.61	60.79
		DCNet	3.10	2.08	1.02	9.31	83.35	92.18	74.69	59.60
		SSFA (Ours)	**1.88**	**1.62**	**0.26**	**8.52**	81.71	91.60	73.80	58.48
CREMI	Fully	UNet	4.29	2.30	1.99	13.30	91.12	97.65	86.66	76.45
		TopoLoss	**3.15**	2.12	**1.02**	15.24	**91.72**	97.60	86.68	76.49
		clDice	3.33	2.03	1.30	13.56	90.60	97.32	85.48	74.63
		MRE	3.37	1.94	1.43	12.91	91.22	97.67	86.82	76.70
	Semi	MT	3.43	**1.90**	1.52	12.94	91.39	**97.68**	**86.84**	**76.74**
		CPS	3.74	1.98	1.76	13.41	90.92	97.61	86.45	76.14
		XNet	3.32	1.96	1.36	**12.90**	90.68	97.67	86.77	76.64
		DCNet	3.41	1.91	1.50	13.27	91.25	97.52	85.99	75.42
		SSFA (Ours)	**2.15**	**1.77**	**0.38**	**11.88**	**91.50**	**97.77**	**87.50**	**77.78**
STARE-DRIVE	Fully	UNet	424.13	6.25	417.88	19.89	61.43	94.69	62.66	45.62
		TopoLoss	371.13	7.91	363.22	**15.36**	66.96	95.39	64.47	47.57
		clDice	452.06	12.34	439.72	22.93	63.46	92.12	61.42	44.33
		MRE	**346.99**	8.93	**338.06**	16.18	67.42	95.06	67.46	50.89
	Semi	MT	381.38	6.19	375.19	18.56	64.30	95.26	65.91	49.15
		CPS	380.97	5.47	375.50	17.22	**68.53**	**95.64**	**69.43**	**53.17**
		XNet	355.91	6.20	349.71	17.84	66.78	95.00	64.40	47.50
		DCNet	365.54	**5.30**	360.24	17.18	67.28	95.28	69.88	53.70
		SSFA (Ours)	**205.56**	**5.22**	**200.34**	**13.44**	**72.91**	**95.83**	**72.20**	**56.49**

a TopoLoss, clDice, and MRE use UNet as the segmentation architecture. MT, CPS, XNet, DCNet, and SSFA are all based on UNet. Red and **bold** indicate the best and second-best performance, respectively.

### 4.5 Ablation studies

To verify the effectiveness of each component, we conduct the following ablation studies on four datasets.

**Comparison of** $\lambda_{\text{max}}$ . The results are shown in Table 3. For smaller datasets (such as ER), a larger $\lambda_{\text{max}}$ is preferable because it accelerates the increase of λ, emphasizing the contribution of unlabeled images and preventing overfitting. For larger datasets (such as SNEMI3D, CREMI and STARE-DRIVE), a smaller $\lambda_{\text{max}}$ is more effective, allowing λ to change gradually. It helps the model leverage labeled images early on and progressively benefit from unlabeled images as training progresses. We ultimately set $\lambda_{\text{max}}$ to 3.0, 1.0, 0.2 and 0.2 for the four datasets and apply these values in all related experiments in Table 2.

**Table 3.** Comparison of different values of $\lambda_{\text{max}}$ on four datasets.^a^

$\lambda_{\text{max}}$	ER								SNEMI3D							
	β ↓	$\beta_{0}\;↓$	$\beta_{1}\;↓$	FR ↓	clDice ↑	Acc ↑	Dice ↑	Jaccard ↑	β ↓	$\beta_{0}\;↓$	$\beta_{1}\;↓$	FR ↓	clDice ↑	Acc ↑	Dice ↑	Jaccard ↑
0.2	24.60	14.80	9.80	**8.81**	94.01	91.30	84.53	73.20	**1.87**	1.63	**0.25**	8.94	81.49	91.49	73.79	58.47
0.5	22.23	**14.20**	8.03	9.27	94.33	91.36	84.36	72.95	1.95	1.66	0.29	9.15	80.58	91.07	72.89	57.34
1.0	23.10	14.88	8.23	9.33	94.68	**91.62**	85.18	74.19	1.88	**1.62**	0.26	**8.52**	**81.71**	**91.60**	**73.80**	**58.48**
3.0	**19.23**	14.65	**4.58**	9.38	**94.71**	91.56	**85.33**	**74.42**	2.09	1.74	0.35	10.42	80.26	91.15	72.81	57.25
5.0	23.70	16.45	7.25	9.91	93.91	91.04	83.57	71.77	2.27	1.89	0.38	10.07	81.26	91.41	73.64	58.28

$\lambda_{\text{max}}$	CREMI								STARE-DRIVE							
	β ↓	$\beta_{0}\;↓$	$\beta_{1}\;↓$	FR ↓	clDice ↑	Acc ↑	Dice ↑	Jaccard ↑	β ↓	$\beta_{0}\;↓$	$\beta_{1}\;↓$	FR ↓	clDice ↑	Acc ↑	Dice ↑	Jaccard ↑
0.2	**2.15**	**1.77**	**0.38**	**11.88**	91.50	97.77	87.50	77.78	**248.47**	4.94	**243.53**	13.98	71.72	95.64	71.49	55.63
0.5	2.20	1.81	0.39	12.01	91.47	97.75	87.41	77.63	258.72	4.84	253.88	**13.93**	**74.32**	**96.02**	**73.79**	**58.46**
1.0	2.22	1.81	0.41	12.12	**91.51**	**97.78**	**87.52**	**77.82**	254.06	6.09	247.97	15.88	71.58	95.74	70.57	54.52
3.0	2.28	1.85	0.43	12.53	91.36	97.61	86.61	76.38	329.91	9.66	320.25	23.65	61.51	94.82	62.61	45.57
5.0	2.30	1.80	0.50	13.37	90.90	97.46	85.73	75.02	338.72	**4.66**	334.06	16.24	68.54	95.37	68.92	52.58

a The FA Encoder uses a multi-layer architecture, and the update weight *k* are set to 0.6, 1.0, 0.4, and 0.0, respectively.

Bold indicate the best performance.

**Comparison of** k . From Table 4, we observe that the model’s performance is relatively stable across different values of k. Generally, larger values of k (ranging from 0.6 to 1.0) lead to improved topological and volumetric performance. We ultimately set k to 0.6, 1.0, 1.0, and 0.6 for the four datasets and apply these values in all related experiments in Table 2.

**Table 4.** Comparison of different values of *k* on four datasets.^a^

*k*	ER								SNEMI3D							
	β ↓	$\beta_{0}\;↓$	$\beta_{1}\;↓$	FR ↓	clDice ↑	Acc ↑	Dice ↑	Jaccard ↑	β ↓	$\beta_{0}\;↓$	$\beta_{1}\;↓$	FR ↓	clDice ↑	Acc ↑	Dice ↑	Jaccard ↑
0.0 (w/o update)	20.45	15.30	5.15	9.43	94.73	**91.59**	85.26	74.31	2.33	1.94	0.39	9.05	81.05	90.13	70.91	54.94
0.2	22.85	15.35	7.50	**9.14**	94.49	91.50	84.90	73.76	1.94	1.66	0.28	8.76	81.29	91.41	73.55	58.16
0.4	20.58	14.70	5.88	9.21	94.60	91.58	85.12	74.09	2.08	1.69	0.39	8.73	**81.87**	91.43	73.95	58.66
0.6	**19.23**	**14.65**	4.58	9.38	94.71	91.56	**85.33**	**74.42**	2.17	1.79	0.38	8.82	81.86	**91.73**	**74.12**	**58.88**
0.8	19.75	15.48	**4.28**	9.45	**94.75**	91.56	85.30	74.37	2.18	1.70	0.49	9.30	81.42	91.29	73.55	58.17
1.0	22.70	16.33	6.38	9.29	94.55	91.50	84.97	73.87	**1.88**	**1.62**	**0.26**	**8.52**	81.71	91.60	73.80	58.48

*k*	CREMI								STARE-DRIVE							
	β ↓	$\beta_{0}\;↓$	$\beta_{1}\;↓$	FR ↓	clDice ↑	Acc ↑	Dice ↑	Jaccard ↑	β ↓	$\beta_{0}\;↓$	$\beta_{1}\;↓$	FR ↓	clDice ↑	Acc ↑	Dice ↑	Jaccard ↑
0.0 (w/o update)	2.24	1.82	0.42	12.43	91.49	97.70	87.20	77.31	248.47	4.94	243.53	13.98	71.72	95.64	71.49	55.63
0.2	2.27	1.82	0.45	12.83	91.34	97.74	87.30	77.46	269.06	**4.88**	264.19	13.90	73.37	95.95	72.65	57.05
0.4	2.22	1.81	0.41	**12.12**	**91.51**	**97.78**	**87.52**	**77.82**	253.31	5.34	247.97	13.46	73.85	96.01	73.12	57.63
0.6	2.22	1.79	0.43	12.41	91.28	97.59	86.53	76.26	**205.56**	5.22	**200.34**	13.44	72.91	95.83	72.20	56.49
0.8	2.20	**1.78**	0.42	13.17	91.11	97.58	86.45	76.13	247.34	5.19	242.16	**13.43**	**74.44**	**96.06**	**74.18**	**58.95**
1.0	**2.18**	1.88	**0.30**	12.81	91.25	97.44	85.88	75.25	278.66	5.13	273.53	14.59	71.76	95.92	71.75	55.95

a The FA Encoder uses a multi-layer architecture, and the maximum weight $\lambda_{\max}$ of the unsupervised loss are set to 3.0, 1.0, 1.0 and 0.2, respectively.

Bold indicate the best performance.

**Effectiveness of FA Map.** To demonstrate the positive impact of the FA Map on reducing fractures, we incorporate it into the semi-supervised model CPS (Chen *et al*. 2021) as an additional input channel. From Table 5, we find that the FA Map significantly enhances topological performance compared to without it. This improvement may be due to the FA Map providing structural prior information, such as thickness and differences, which helps the model focus more effectively on fracture-prone structures during training.

**Table 5.** Comparison of CPS with and without the FA Map on four datasets.^a^

FA Map	ER								SNEMI3D							
	β ↓	$\beta_{0}\;↓$	$\beta_{1}\;↓$	FR ↓	clDice ↑	Acc ↑	Dice ↑	Jaccard ↑	β ↓	$\beta_{0}\;↓$	$\beta_{1}\;↓$	FR ↓	clDice ↑	Acc ↑	Dice ↑	Jaccard ↑
w/o	33.55	18.08	15.48	10.37	93.84	91.10	83.72	71.99	2.71	1.90	0.81	**8.71**	**84.57**	**92.47**	**76.13**	**61.45**
w/	**19.35**	**15.38**	**3.98**	**9.49**	**94.17**	**91.17**	**84.11**	**72.58**	**2.06**	**1.75**	**0.31**	9.37	81.45	90.95	73.13	57.64

FA Map	CREMI								STARE-DRIVE							
	β ↓	$\beta_{0}\;↓$	$\beta_{1}\;↓$	FR ↓	clDice ↑	Acc ↑	Dice ↑	Jaccard ↑	β ↓	$\beta_{0}\;↓$	$\beta_{1}\;↓$	FR ↓	clDice ↑	Acc ↑	Dice ↑	Jaccard ↑
w/o	3.74	1.98	1.76	13.41	90.92	97.61	86.45	76.14	380.97	**5.47**	375.50	17.22	68.53	95.64	69.43	53.17
w/	**2.15**	**1.81**	**0.34**	**11.96**	**91.50**	**97.79**	**87.54**	**77.84**	**243.44**	6.38	**237.06**	**14.98**	**72.51**	**95.71**	**71.50**	**55.64**

a w/o indicates results without the FA Map (using only raw images as input) while w/indicates results with the FA Map as an additional input channel.

Bold indicate the best performance.

**Effectiveness of FA Encoder.** Table 6 shows the performance of different FA Encoder architectures. Incorporating the FA Encoder achieves improved performance compared to using only Raw Image Encoder. Additionally, the multi-layer FA Encoder outperforms the single-layer encoding structure. We ultimately use the multi-layer FA Encoder in all related experiments in Table 2.

**Table 6.** Comparison of different architectures for the FA Encoder on four datasets.^a^

FA Encoder	ER								SNEMI3D							
	β ↓	$\beta_{0}\;↓$	$\beta_{1}\;↓$	FR ↓	clDice ↑	Acc ↑	Dice ↑	Jaccard ↑	β ↓	$\beta_{0}\;↓$	$\beta_{1}\;↓$	FR ↓	clDice ↑	Acc ↑	Dice ↑	Jaccard ↑
Channel	19.35	15.38	**3.98**	9.49	94.17	91.17	84.11	72.58	2.06	1.75	0.31	9.37	81.45	90.95	73.13	57.64
Single	24.83	15.08	9.75	9.61	93.61	90.95	83.31	71.40	2.09	1.83	**0.26**	9.22	81.65	90.99	72.85	57.30
Multiple	**19.23**	**14.65**	4.58	**9.38**	**94.71**	**91.56**	**85.33**	**74.42**	**1.88**	**1.62**	0.26	**8.52**	**81.71**	**91.60**	**73.80**	**58.48**

FA Encoder	CREMI								STARE-DRIVE							
	β ↓	$\beta_{0}\;↓$	$\beta_{1}\;↓$	FR ↓	clDice ↑	Acc ↑	Dice ↑	Jaccard ↑	β ↓	$\beta_{0}\;↓$	$\beta_{1}\;↓$	FR ↓	clDice ↑	Acc ↑	Dice ↑	Jaccard ↑
Channel	2.15	1.81	**0.34**	11.96	**91.50**	**97.79**	**87.54**	**77.84**	243.44	6.38	237.06	14.98	72.51	95.71	71.50	55.64
Single	**2.13**	1.78	0.35	12.49	91.08	97.44	85.82	75.17	233.34	5.65	227.69	13.89	**75.48**	**96.19**	**74.28**	**59.08**
Multiple	2.15	**1.77**	0.38	**11.88**	**91.50**	97.77	87.50	77.78	**205.56**	**5.22**	**200.34**	**13.44**	72.91	95.83	72.20	56.49

a Single indicates a single-layer encoding structure that performs one element-wise multiplication. Multiple indicates a multi-layer encoding structure. Channel indicates the FA Map is used as an additional input channel to the Raw Image Encoder.

Bold indicate the best performance.

## 5 Qualitative results

**Figure 6.**
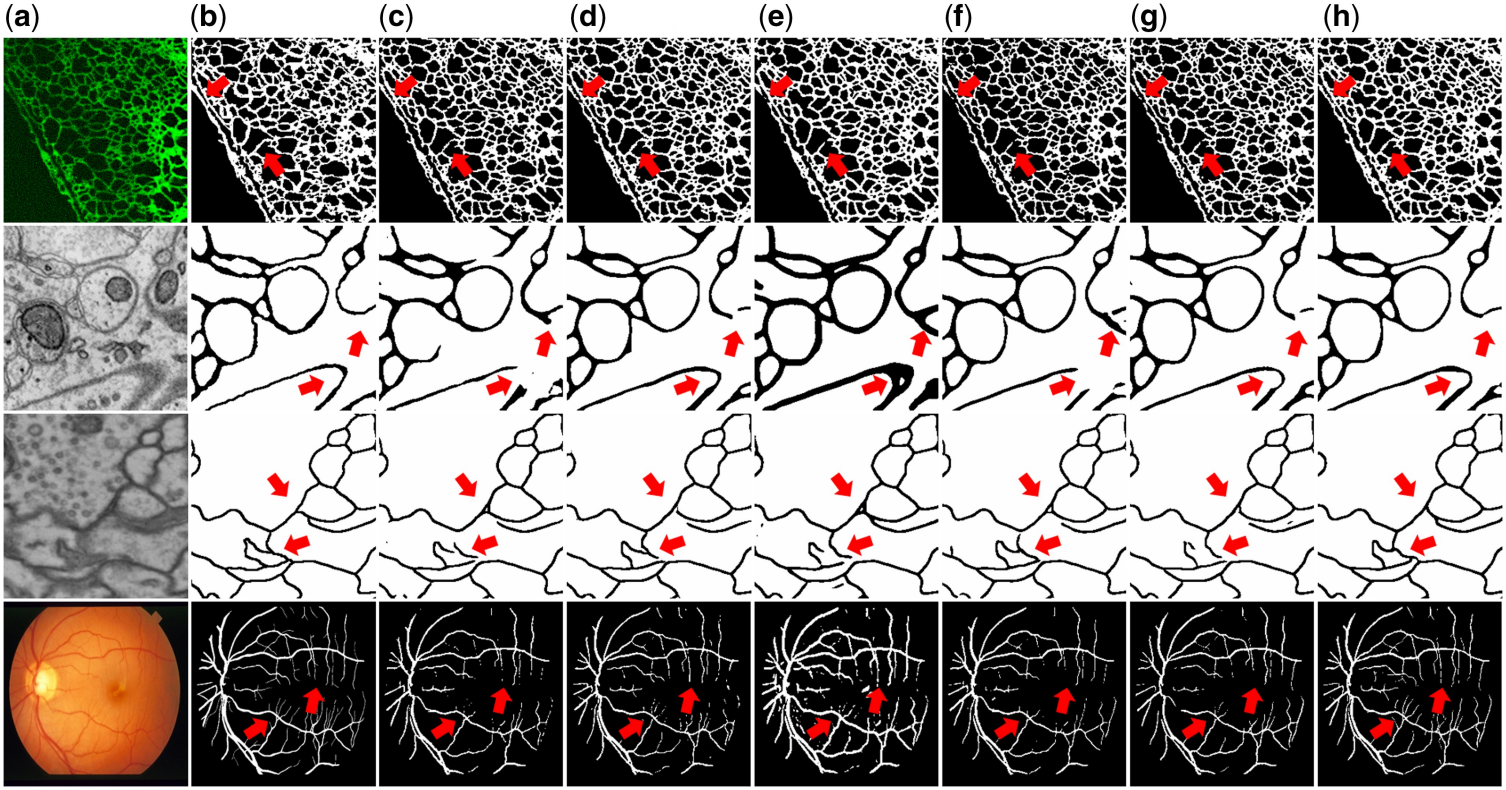
Qualitative results on ER, SNEMI3D, CREMI, and STARE-DRIVE. (a) Raw images. (b) Ground truth. (c) UNet. (d) TopoLoss. (e) clDice. (f) MT. (g) CPS. (h) SSFA. Red arrows highlight differences among the results.

Figure 6 shows some qualitative results from different fully and semi-supervised models. By introducing the TS Map and DS Map, our model more effectively focuses on slender and narrow branches, as well as semantically ambiguous regions. It significantly reduces fractures in the segmentation results.

## 6 Conclusion

We propose the fracture-attention model, SSFA, for semi-supervised segmentation of tubular objects. SSFA achieves state-of-the-art performance in topological accuracy while maintaining volumetric precision. Our model utilizes the FA Map as an additional input to focus on fracture-prone structures and employs the FA Encoder to extract structural information, which enhances connectivity in the segmentation results. Additionally, we introduce an intuitive metric to evaluate the FR in segmentation results. Extensive experiments on four datasets demonstrate the effectiveness of our model.

## References

[btaf013-B1] Banerjee S , Magee L, Wang D et al Semantic segmentation of microscopic neuroanatomical data by combining topological priors with encoder–decoder deep networks. Nat Mach Intell 2020;2:585–94. 34604701 10.1038/s42256-020-0227-9 PMC8486300

[btaf013-B2] Belli D , Kipf T. Image-conditioned graph generation for road network extraction. arXiv: 1910.14388, 2019, preprint: not peer reviewed.

[btaf013-B3] Chen F , Fei J, Chen Y et al Decoupled consistency for semi-supervised medical image segmentation. In: *MICCAI*. Vancouver, Canada: Springer, 2023, 551–561.

[btaf013-B4] Chen X , Yuan Y, Zeng G et al Semi-supervised semantic segmentation with cross pseudo supervision. In: *CVPR*, Nashville, TN, USA, 2021, 2613–2622.

[btaf013-B5] Dulau I , Helmer C, Delcourt C et al Ensuring a connected structure for retinal vessels deep-learning segmentation. In: *ICCV*, Paris - France, 2023, 2364–2373.

[btaf013-B6] Funke J , Saalfeld S, Bock D et al *Miccai Challenge on Circuit Reconstruction from Electron Microscopy Images*. In: MICCAI. Springer, 2016.

[btaf013-B7] Hoover A , Kouznetsova V, Goldbaum M. Locating blood vessels in retinal images by piecewise threshold probing of a matched filter response. IEEE Trans Med Imaging 2000;19:203–10. 10875704 10.1109/42.845178

[btaf013-B8] Hu J , Shen L, Sun G. Squeeze-and-excitation networks. In: *CVPR*, Salt Lake City, UT, USA, 2018, 7132–7141.

[btaf013-B9] Hu X , Li F, Samaras D et al Topology-preserving deep image segmentation. In: NeurIPS, Vancouver, BC, Canada, Vol. 32, 2019.

[btaf013-B10] Huang J , Luo Y, Guo Y et al Accurate segmentation of intracellular organelle networks using low-level features and topological self-similarity. Bioinformatics 2024;40:btae559. 39302662 10.1093/bioinformatics/btae559 PMC11467052

[btaf013-B11] Luo Y , Guo Y, Li W et al Fluorescence microscopy image datasets for deep learning segmentation of intracellular orgenelle networks. In: *Dataport*. IEEE, 2020. 10.21227/t2he-zn97

[btaf013-B12] Milletari F , Navab N, Ahmadi S-A. V-net: Fully convolutional neural networks for volumetric medical image segmentation. In: *3DV*, Stanford, California, USA: IEEE, 2016, 565–571.

[btaf013-B13] Mosinska A , Marquez-Neila P, Koziński M et al Beyond the pixel-wise loss for topology-aware delineation. In: *CVPR*, Salt Lake City, UT, USA, 2018, 3136–3145.

[btaf013-B14] Ngoc MÔV , Chen Y, Boutry N et al Introducing the boundary-aware loss for deep image segmentation. In: BMVC, Online, 2021.

[btaf013-B15] Qi Y , He Y, Qi X et al Dynamic snake convolution based on topological geometric constraints for tubular structure segmentation. In: *ICCV*, Paris, France, 2023, 6070–6079.

[btaf013-B16] Ronneberger O , Fischer P, Brox T. U-net: Convolutional networks for biomedical image segmentation. In: *MICCAI*. Springer, Munich, Germany, 2015, 234–241.

[btaf013-B17] Shit S , Paetzold JC, Sekuboyina A et al cldice-a novel topology-preserving loss function for tubular structure segmentation. In: *CVPR*, Nashville, TN, USA, 2021, 16560–16569.

[btaf013-B18] Simonyan K , Zisserman A. Very deep convolutional networks for large-scale image recognition. arXiv:1409.1556, 2014, preprint: not peer reviewed.

[btaf013-B19] SNEMI3D, Challenge. *Isbi 2013 challenge: 3d segmentation of neurites in em images*. 2017. 2013 (January 1, 2020, date last accessed). https://snemi3d.grand-challenge.org/

[btaf013-B20] Staal J , Abràmoff MD, Niemeijer M et al Ridge-based vessel segmentation in color images of the retina. IEEE Trans Med Imaging 2004;23:501–9. 15084075 10.1109/TMI.2004.825627

[btaf013-B21] Tarvainen A , Valpola H. Mean teachers are better role models: weight-averaged consistency targets improve semi-supervised deep learning results. In: NeurIPS, Long Beach, CA, USA, Vol. 30, 2017.

[btaf013-B22] Van Etten A , Lindenbaum D, Bacastow TM. Spacenet: A remote sensing dataset and challenge series. arXiv:1807.01232, 2018, preprint: not peer reviewed.

[btaf013-B23] Yang J , Hu X, Chen C et al 3d topology-preserving segmentation with compound multi-slice representation. In: *ISBI*. Online: IEEE, 2021, 1297–1301.

[btaf013-B24] Zhou Y , Huang J, Wang C et al Xnet: Wavelet-based low and high frequency fusion networks for fully- and semi-supervised semantic segmentation of biomedical images. In: *ICCV*, Paris, France, 2023, 21085–21096.

